# *Culex pipiens* forms and urbanization: effects on blood feeding sources and transmission of avian *Plasmodium*

**DOI:** 10.1186/s12936-016-1643-5

**Published:** 2016-12-08

**Authors:** Josué Martínez-de la Puente, Martina Ferraguti, Santiago Ruiz, David Roiz, Ramón C. Soriguer, Jordi Figuerola

**Affiliations:** 1 Estación Biológica de Doñana (EBD-CSIC), Avda Américo Vespucio s/n, 41092 Seville, Spain; 2 Servicio de Control de Mosquitos, Diputación de Huelva, Huelva, Spain; 3 CIBER Epidemiología y Salud Pública (CIBERESP), Madrid, Spain; 4 Infectious Diseases and Vectors: Ecology, Genetics, Evolution and Control, IRD (Institut de Recherche pour le Développement), Montpellier, France

**Keywords:** Avian malaria, Birds, Diseases, *Haemoproteus*, Humans, Mosquitoes, Pathogens, Vector-borne diseases, Wildlife

## Abstract

**Background:**

The wide spread mosquito *Culex pipiens pipiens* have two forms *molestus* and *pipiens* which frequently hybridize. The two forms have behavioural and physiological differences affecting habitat requirements and host selection, which may affect the transmission dynamic of *Cx. p. pipiens*-borne diseases.

**Methods:**

During 2013, blood engorged *Cx. p. pipiens* mosquitoes were captured in urban, rural and natural areas from Southern Spain. In 120 mosquitoes, we identified the blood meal origin at vertebrate species/genus level and the mosquito form. The presence and molecular lineage identity of avian malaria parasites in the head-thorax of each mosquito was also analysed.

**Results:**

Mosquitoes of the form *pipiens* were more frequently found in natural than in urban areas. The proportion of *Cx. pipiens* form *molestus* and hybrids of the two forms did not differ between habitat categories. Any significant difference in the proportion of blood meals on birds between forms was found. Birds were the most common feeding source for the two forms and their hybrids. Among mammals, dogs and humans were the most common hosts. Two *Plasmodium* and one *Haemoproteus* lineages were found in mosquitoes, with non-significant differences between forms.

**Conclusion:**

This study supports a differential distribution of *Cx. p. pipiens* form *pipiens* between urban and natural areas. Probably due to the similar feeding sources of both mosquito forms and their hybrids here, all of them may frequently interact with avian malaria parasites playing a role in the transmission of *Plasmodium*.

## Background

The *Culex pipiens* complex include species such as the common house mosquito *Culex pipiens pipiens* that is a wide spread species distributed over extensive areas in Europe and Africa and has been introduced in the Americas and in some temperate areas of Asia and Australia [1, 2]. Two forms or biotypes have been described: the *molestus* and the *pipiens* forms [1]. Although morphologically indistinguishable as adults, these two forms present genetic, behavioural and physiological differences. The *molestus* form is stenogamous and autogenous, that is mosquitoes are able to mate in confined environments and lay their eggs in absence of a previous blood meal, respectively. By contrast, mosquitoes of the *pipiens* form use open environments for mating (eurygamous) and requires a blood meal, as a nutrient source, for oviposition (anautogenous). In North European countries, the two forms use different habitats with *Cx. p. pipiens* form *molestus* frequently living in underground environments in areas with human influence, while mosquitoes of the *pipiens* form are mainly present in aboveground habitats [3, 4]. In countries of the Mediterranean basin, mosquitoes of the two forms are sympatric and, because *molestus* and *pipiens* forms are not completely genetically isolated, hybrids are frequent [5–8]. Hybridization between the two forms has been also occasionally reported in North Europe, in Germany [9], Austria [10], the Netherlands [11, 12] and the UK [13]. Hybridization between forms has also been reported under laboratory conditions [14].

The importance of mosquito species for the transmission of vector-borne pathogens is strongly determined by, among other factors, their feeding behaviour [15]. In addition to the differential competence of each mosquito species to develop a particular pathogen, the feeding behaviour of mosquitoes determines the contact rate between infected and susceptible vertebrate hosts. *Culex p. pipiens* play a key role in the transmission of numerous vector borne pathogens affecting humans, livestock and wildlife including viruses (e.g. West Nile virus, WNV; [16]), protozoa (e.g. avian malaria; [17]) and metazoa (e.g. filarial worms [18]) parasites (reviewed by [2]). *Culex p. pipiens* females feed mainly on birds (69–97%) [19], but mammals are also an important fraction of their blood meals, compromising over 20% of the blood meals in some populations [19, 20], also see [2, 21, 22]. However, it has been suggested that *Cx. p. pipiens* form *molestus* feed mainly on mammals while *Cx. p. pipiens* form *pipiens* feed mainly on birds [23], see also [24]. Hybrids show a higher vectorial competence for the transmission of some pathogens than both *pipiens* and *molestus* forms (e.g. WNV, [25]), and it has been suggested that due to their intermediate feeding behaviour [23], they may act as bridge vectors for the transmission of pathogens between birds and humans, as in the case of WNV [4].

Avian malaria parasites of the genus *Plasmodium* are mosquito-borne parasites commonly found infecting birds [26]. Nowadays, approximately 40 different species have been described [26] but, since the seminar paper by Bensch et al. [27], a high genetic diversity of cytochrome *b* (cyt *b*) lineages of *Plasmodium* parasites have been identified. Several mosquito genera transmit avian malaria parasites, including different species of the genus *Culex,* which may play a central role in the transmission of avian malaria [17]. In particular, *Cx. p. pipiens* may play a key role in the transmission dynamic of *Plasmodium* parasites under natural conditions. *Plasmodium* infected birds are common hosts of this mosquito species, allowing frequent parasite-*Cx. p. pipiens* encounters [22, 28]. Furthermore, avian *Plasmodium* parasites, including *P. relictum*, successfully develops in *Cx. p. pipiens* as supported by experimental infections of mosquitoes [17, 29–31], and different avian *Plasmodium* lineages have been molecularly isolated from mosquitoes trapped in the wild [32–37].

The aims of this study were (1) to investigate the spatial distribution of *pipiens* and *molestus* forms and their hybrids in relation to the type of habitats (natural, rural and urban areas), (2) to compare the feeding patterns of both forms in these habitats and (3) to determine avian malaria prevalence and diversity in these mosquitoes. Previous evidence suggest that the two *Cx. p. pipiens* forms and their hybrids use differential habitats [3, 10], thus a higher frequency of the *molestus* form is expected in urban areas than in natural ones, while the opposite pattern is expected for mosquitoes of the *pipiens* form. Furthermore, the *molestus* form may feed mainly on mammals, blood meals from mosquitoes of the *pipiens* form should be predominantly avian-derived [38] while their hybrids may show an intermediate feeding behaviour [23, 38]. If the feeding pattern differs between mosquito forms, the prevalence of infection by avian malaria parasites will be higher in the *pipiens* form due to the expected higher frequency of avian-derived blood meals with a decreased prevalence in mosquitoes of the *molestus* form and hybrids as the proportion of mammal-derived blood meals increased.

## Methods

**Fig. 1:**
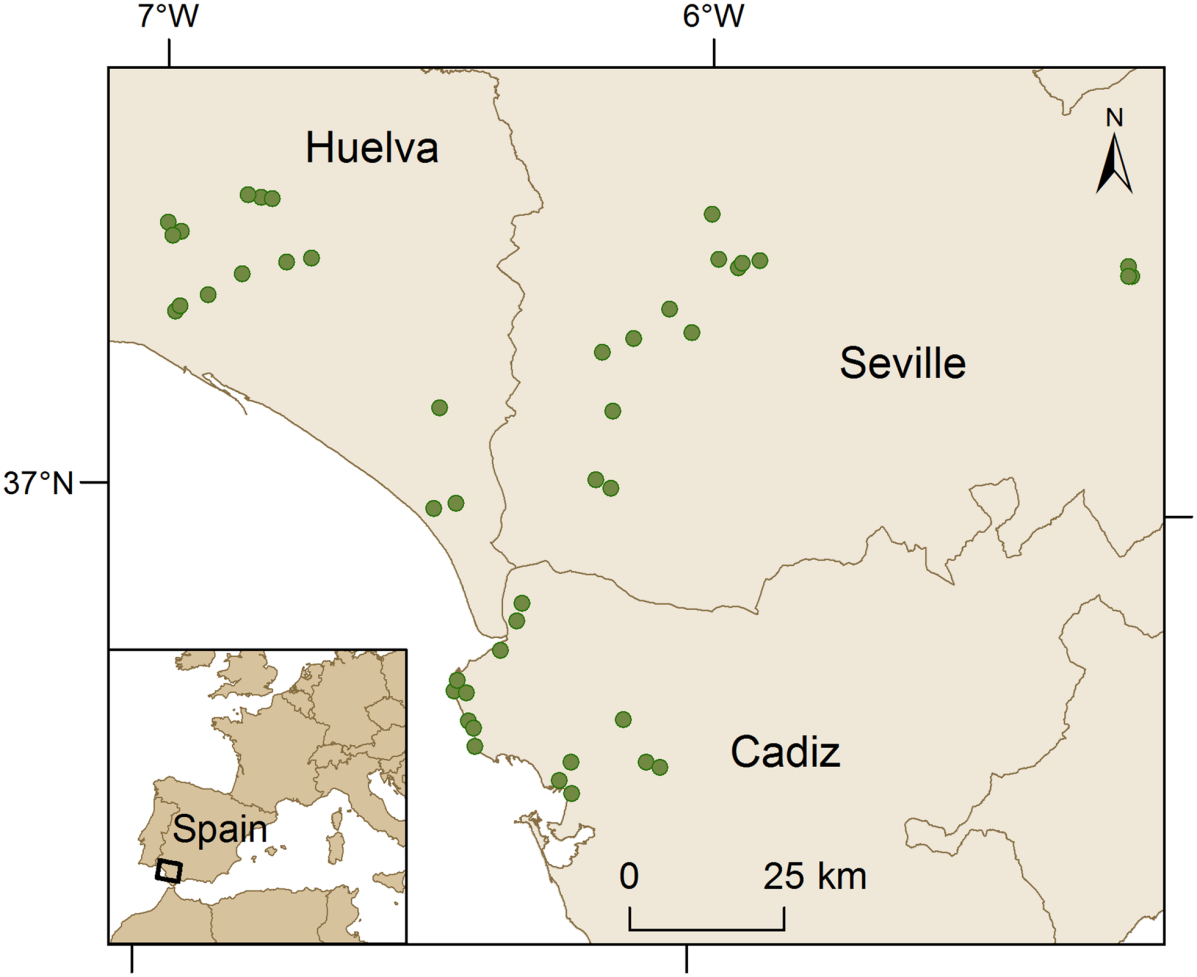
Distribution of the 45 mosquito sampling sites in Southern Spain

Mosquitoes were captured using BG-sentinel traps baited with BG-lure and dry ice as a source of CO_2_. Overall, from April to December, 5–6 trapping sessions were conducted at each site. In each trapping session, three traps were operated for 24 h at 45 different sampling sites in Cadiz, Huelva and Seville provinces (15 per each province) once every 45 days (Fig. 1). The sampling sites were grouped in triplets including one natural habitat (conserved landscapes with low density of human and livestock), one rural habitat (farms with livestock) and one urban habitat (urbanized densely populated areas), see [39] for further details of sampling protocol.

Adult mosquitoes were preserved in dry ice and stored frozen until morphological identification. Mosquitoes were sorted on a Petri plate on a chill table under a stereomicroscope and morphologically identified to species level following [40, 41]. *Culex p. pipiens* females with a recent blood meal in the abdomen were selected for this study. The abdomen of engorged mosquitoes was separated from the head-thorax using sterile tips. Genomic DNA of each the abdomen and the head-thorax of each mosquito was extracted using the DNA Kit Maxwell^®^ 16LEV kit [42].

The DNA extracted from the mosquito abdomen was employed to identify the vertebrate origin of the blood meals using a nested PCR following Alcaide et al. [43]. Briefly, this method amplifies a 758-base pairs fragment (excluding primers) of the mitochondrial cytochrome oxidase 1 (COI) gene (barcoding region) of vertebrate species. Amplicons were sequenced in the Macrogen sequencing service (Macrogen Inc., The Netherlands). Vertebrate hosts of mosquitoes were identified by comparisons of the sequences obtained from mosquitoes with those available in public databases (GenBank DNA sequence database, National Center for Biotechnology Information Blast, and/or the Barcode of Life Data Systems).

The DNA extracted from the head-thorax of each engorged mosquito was used to identify the *Cx. p. pipiens* form and the presence and identity of avian malaria parasite lineages. The mosquito forms were identified following Bahnck and Fonseca [44]. This method is based in the amplification of the 5′ flacking region of CQ11 microsatellite and has been routinely used to identify the *Cx. p. pipiens* forms in Old-world countries [5, 7, 8], including Spain [45]. The presence and identity of *Plasmodium* and *Haemoproteus* parasites in the head-thorax of mosquitoes was determined following Helgreen et al. [46]. Parasite lineages were determined by BLAST comparison of the sequences with those deposited in Genbank and the morphospecies was determined based on the information available in MalAvi [47].

Statistical analyses were conducted using the GLIMMIX procedure in SAS 9.2 with a binomial distribution using a Laplace approximation. Firstly, generalized linear mixed models were used to identify potential differences in the occurrence of each form (*molestus* or *pipiens*) and their hybrids between habitats. The triplet (see sampling sites above) was included as a random factor in order to control for geographical pseudo-replication of the samples. Secondly, differences in the presence of avian-derived blood meals between mosquito forms were tested using the presence/absence of avian derived blood meals as the dependent variable, mosquito forms and habitat category as fixed factors and triplet as a random factor. Finally, differences in the prevalence of blood parasites between mosquito forms and habitats were tested by including parasite infection status as the dependent variable, mosquito forms and habitat category as fixed factors and triplet as a random factor.

## Results

**Fig. 2:**
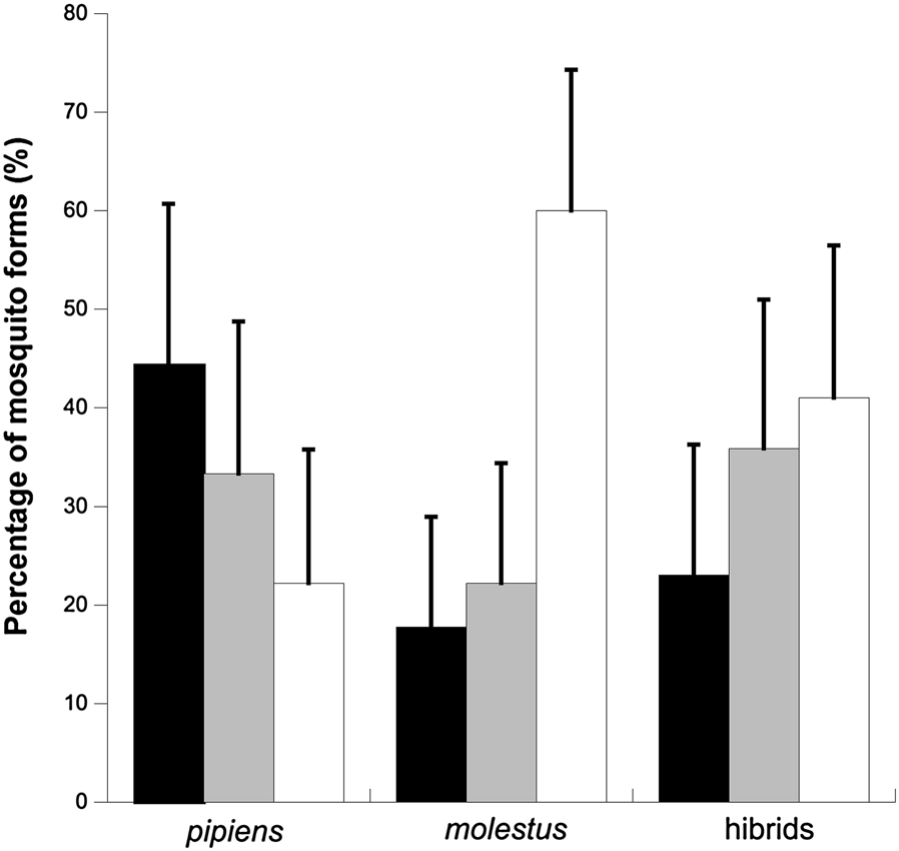
Percentage of mosquitoes of the form *pipiens,* form *molestus* and their hybrids found in natural (*black*), rural (*grey*) and urban (*white*) areas in Southern Spain. *Bars* indicate 95% confidence intervals

Overall, the vertebrate origin of 120 mosquito blood meals was identified to the species/genus level, corresponding to 33, 36, 51 individuals trapped in natural, rural and urban areas, respectively. Frequency of mosquitoes of the *pipiens* form differed between habitats (Fig. 2; F_2,103_ = 3.96; p = 0.02) being significantly (t = 32.77; p < 0.01) more frequently found in natural (44%, 16/36) than in urban areas (22%, 8/36). Mosquitoes of the *molestus* form were more frequently found in urban (60%, 27/45) than in natural areas (18%, 8/45), although differences among habitat categories did not reach significance (Fig. 1; F = 2.19; df = 2,103; p = 0.12). Both *molestus* (22%, 10/45) and *pipiens* (33%, 12/36) forms showed intermediate percentages in rural environments. Hybrids were similarly distributed between habitat categories (Fig. 2; F_2,103_ = 0.55; p = 0.58; natural: 23%, 9/39; rural: 36%, 14/39; urban: 41%, 16/39).

Most of the blood meals derived from birds (n = 80, 66.7%), while only 40 mosquitoes fed on mammal blood (33.3%) (Table 1). The vertebrate hosts identified from mosquitoes included, at least, 20 bird and 9 mammal species. House sparrows and dogs were the most common bird and mammal hosts of mosquitoes, respectively. Moreover, humans were identified as hosts of *pipiens* and *molestus* forms and their hybrids (Table 1). The presence of avian-derived blood meals did not differ between forms nor habitats (mosquito form: F_2,101_ = 0.16; p = 0.85; habitat: F_2,101_ = 1.98; p = 0.14).

**Table 1:** Number of mosquitoes with blood meals from different avian and mammal host species

	*Pipiens* from	*Molestus* form	Hybrids	Total
*Alectoris rufa*	1	1		2
*Anas crecca*	1			1
*Anthus pratensis*		1		1
*Ardeola ralloides*	1			1
*Asio otus*		1		1
*Bubulcus ibis*			1	1
*Burhinus oedicnemus*		1		1
*Carduelis chloris*			2	2
*Columba livia*			1	1
*Galerida cristata*	1			1
*Gallus gallus*	3	5	4	12
*Hippolais polyglotta*			1	1
*Ixobrychus minutus*		1		1
*Larus ridibundus*		1		1
*Nycticorax nycticorax*			1	1
*Passer domesticus*	6 (1)	8 (1)	9	23
*Streptopelia decaocto*	1	5	2	8
*Sturnus* sp.	3	1 (1)		4
*Sylvia melanocephala*	2 (1)	2	1	5
*Sylvia* sp.		2		2
*Turdus merula*	5 (3)	3	2	10
Total birds	24	32	24	80
*Bos taurus*	2			2
*Canis lupus familiaris*	5	6	10 (1)	21
*Equus caballus*		1	2	3
*Felis silvestris*	1	1		2
*Homo sapiens*	2	3	1	6
*Lepus granatensis*	1			1
*Oryctolagus cuniculus*		1		1
*Rattus rattus*		1	1	2
*Rattus* sp.	1			1
*Sus scrofa*			1	1
Total mammals	12	13	15	40
Total	36	45	39	120

The number of mosquitoes carrying parasites is shown between brackets

Parasites were isolated from the head-thorax of eight mosquito females (overall prevalence 6.7%). Two *Plasmodium* lineages were isolated from seven mosquitoes including the cosmopolitan lineage SGS1 corresponding to *Plasmodium relictum* (n = 2, overall prevalence: 1.7%) and the lineage SYAT05 (=Rinshi-11) corresponding to *Plasmodium vaughani* (n = 5; overall prevalence: 4.2%). The *Haemoproteus* lineage Padom05 belonging to *Haemoproteus passeris* was isolated from one mosquito of the form *pipiens* with a house sparrow derived blood meal (Table 2; overall prevalence: 0.8%). The prevalence of blood parasites did not significantly differ between mosquito forms (mosquito form: F_2,101_ = 0.96; p = 0.39; habitat: F_2,101_ = 1.51; p = 0.23), although the absence of significant differences could be due to the low prevalence and the low number of mosquitoes analysed.

**Table 2:** Parasite lineages isolated from the head-thorax of *Culex p. pipiens* females with respect to the forms and the blood meal source identified in their abdomen

Parasite lineage	Mosquito form	Blood meal origin
*Haemoproteus* PADOM05	*Pipiens*	*Passer domesticus* (1)
*Plasmodium* SYAT05	*Pipiens*	*Turdus merula* (3)
	*Molestus*	*Sturnus* sp. (1)
	Hybrid	*Canis lupus familiaris* (1)
*Plasmodium* SGS1	*Pipiens*	*Sylvia melanocephala* (1)
	*Molestus*	*Passer domesticus* (1)

The number of mosquitoes identified corresponding to each parasite lineage, form and vertebrate host are shown in brackets

## Discussion

A higher percentage of the *pipiens* form was found in natural than in urban areas, while the percentage of mosquitoes of the *molestus* form and hybrids did not differ between the three habitat categories considered in this study. Traditionally, anautogenous mosquitoes, a characteristic typically assigned to the form *pipiens*, are more commonly found in aboveground and rural habitats, while the autogenous form, corresponding to *Cx. p. pipiens* form *molestus*, is usually found in underground urban environments [1, 4, 48, 49]. However, reports of the differential distribution of genetically characterized mosquito forms in areas with different degrees of anthropization are scarce, especially in Southern Europe. In Portugal, the proportion of mosquitoes of the *pipiens* form was higher in farms located in peri-urban areas without dwellings in the vicinity than indoors in residential areas, supporting a negative association between the frequency of this form and the degree of urbanization [38]. In addition, although the frequency of *molestus* forms did not significantly differ between urban and peri-urban habitats, the proportion of *molestus* mosquitoes tended to be higher in indoors traps in areas densely populated by humans rather than in small villages, suggesting the higher presence of this form in urbanized environments [38]. Our results support those from Osorio et al. [38], in spite of the differences between studies regarding the sampling locations (indoors/outdoors).

It is generally assumed that *pipiens* and *molestus* forms have a bird and mammal blood feeding preference respectively, while hybrids show an intermediate behaviour [23]. This fact suggest that hybrids may play a central role in the transmission of pathogens such as WNV and USUTU virus (USUV), which circulate between birds but occasionally infect humans. In North America, mosquitoes with a higher ancestry from the *molestus* form feed more frequently on mammals, including humans [24, 50]. Contrary to this pattern, we did not find any significant association between mosquito forms and their feeding sources. The two forms and their hybrids feed mainly on birds, with a relative high percentage (33%) of blood meals derived from mammals, including humans. A similar feeding pattern of *Cx. p. pipiens* was reported in Portugal, where mosquitoes of the two forms feed mainly on birds with not significant differences between forms [51], but see [38]. One potential explanation is the different approaches used to identify the mosquito forms between studies, while studies from North America genotyped mosquitoes using seven [50] or ten [24] microsatellite markers, here the mosquito forms were assessed with the CQ11 microsatellite. However, Gomes et al. [51] used six microsatellites and obtained very similar results to our study. Alternatively, mosquito feeding pattern is geographically-dependent [1, 21] and varies seasonally [50, 52]. This variability between areas and seasons may partially explain discrepancies in the feeding source of mosquito forms between studies. The similar feeding behaviour of the two mosquito forms and their hybrids found here supports that all of them could be involved in the transmission of pathogens from birds to humans, as WNV or USUV.

Overall, 6.7% of mosquitoes harboured blood parasites in this study. Glaizot et al. [33] found a similar prevalence (6.6%) in *Culex pipiens*, although slightly higher values were reported in other previous studies [36, 37]. Differences between studies could be due to the temporal variation in the prevalence of *Plasmodium* usually found in mosquitoes [35, 36], but also due to methodological differences between studies including the protocol used for parasite detection (e.g. using engorged mosquitoes or mosquitoes with a completely digested blood meal) or the method used for mosquito sampling [53]. Furthermore, the number of mosquitoes with a mammal and avian derived blood meal may differ between trapping methods [54] potentially biasing estimates of parasite prevalence. This fact should be take into account in studies on the epidemiology of vector-borne parasites transmitted by mosquitoes with an opportunistic feeding behaviour. Nevertheless, in this study, the prevalence of avian malaria parasites did not differ between the two forms and their hybrids, which may be due at least in part, to the similar blood feeding behaviour reported between them. However, the absence of significant differences between forms could also be due to the low statistical power owing to the number of mosquitoes analysed and the low prevalence of blood parasites. Avian *Plasmodium* is a mosquito-borne parasite while the related *Haemoproteus* is mainly transmitted by *Culicoides* and louse flies [26]. As occurred here, previous studies have reported the presence of *Haemoproteus* in mosquitoes [22, 34, 35, 37, 55], however experimental evidence available exclude mosquitoes as vectors of this parasite genus [31, 56]. The *Haemoproteus passeris* lineage Padom05 was isolated from a mosquito with a recent blood meal on a house sparrow, which is a common avian host of this parasite lineage [31, 57]. This, together with the fact that the mosquito had a recent blood meal from this bird species, suggests that the *Haemoproteus* DNA derived from rests of undigested blood present in the head-thorax of the mosquito. Alternatively, it is possible that a non-infective form of the parasite present in the mosquito head-thorax was amplified [56], but see [31]. Although molecular isolation does not demonstrate vector competence, the *Plasmodium* parasites isolated from mosquitoes suggest that the two mosquito forms and their hybrids may frequently interact with birds infected by avian malaria parasites. Moreover, the *Plasmodium* lineages found in this study have been previously isolated from *Cx. pipiens* mosquitoes in Europe including Switzerland [33, 36], Portugal [58], Spain [35], France [37] and Italy [22]. Avian *Plasmodium* develops in laboratory reared *Cx. p. pipiens* form *molestus* [29, 30] and these parasites have been molecularly identified in field-collected mosquitoes of this form [32]. Complete development of avian *Plasmodium* has been reported in mosquitoes of the *pipiens* form [17]. However, to our knowledge, this is the first study testing the potential differences of the two *Cx. p. pipiens* forms and their hybrids in the prevalence of avian *Plasmodium* under natural conditions. Results from this study suggest that, given the similar feeding behaviour of mosquito forms in the study area, the two mosquito forms and their hybrids are similarly involved in the transmission of avian *Plasmodium* in Southern Spain.

## Conclusion

This study supports a differential frequency of mosquitoes of the *pipiens* form between urban and natural areas. While the hypothesis that both forms and their hybrids differ in their feeding sources was not supported by results from this study, with birds as the most common blood source found in all the cases. Consequently, *Cx. p. pipiens molestus* and *pipiens* mosquitoes and their hybrids may frequently interact with WNV, USUV and/or avian malaria parasites playing a role in the transmission of these pathogens.
